# Protective effects and immunomodulation on piglets infected with rotavirus following resveratrol supplementation

**DOI:** 10.1371/journal.pone.0192692

**Published:** 2018-02-21

**Authors:** Qiankun Cui, Qiuting Fu, Xinghong Zhao, Xu Song, Jiankang Yu, Yi Yang, Kai Sun, Lu Bai, Ye Tian, Shufan Chen, Renyong Jia, Yuanfeng Zou, Lixia Li, Xiaoxia Liang, Changliang He, Lizi Yin, Gang Ye, Cheng Lv, Guizhou Yue, Zhongqiong Yin

**Affiliations:** 1 Natural Medicine Research Center, College of Veterinary Medicine, Sichuan Agricultural University, Chengdu, Sichuan, China; 2 Key Laboratory of Animal Disease and Human Health of Sichuan Province, Sichuan Agricultural University, Chengdu, Sichuan, China; 3 College of Life Science, Sichuan Agricultural University, Chengdu, Sichuan, China; University of Liverpool, UNITED KINGDOM

## Abstract

Rotavirus (RV), belonging to *Reoviridae* family, is the leading cause of acute severe viral diarrhea in children (under 5 years old) and infant animals worldwide. Although vaccines are commonly used to prevent infection, episodes of diarrhea caused by RV frequently occur. Thus, this study was conducted to determine whether resveratrol had protective effects against RV infection in piglets. Following pretreatment with resveratrol dry suspension through adding into the basal diet for 3 weeks, the piglets were orally challenged with RV. We found that resveratrol could alleviate diarrhea induced by RV infection. Resveratrol-treatment inhibited the TNF-α production, indicating that the anti-RV activity of resveratrol may be achieved by reducing the inflammatory response. The IFN-γ level was elevated in 10mg/kg/d resveratrol-treated group and 30mg/kg/d resveratrol-treated group after RV infection. The ratios of CD4+/CD8+ in resveratrol-treated groups were the same as that in mock infected group, suggesting that resveratrol could maintain the immune function in RV-infected piglets. It was found that resveratrol could alleviate diarrhea induced by RV infection. These results revealed that resveratrol dry suspension could be a new control measure for RV infection.

## 1. Introduction

Rotavirus (RV) is a double-stranded RNA virus within the family *Reoviridae*. Gastroenteritis induced by RV is the foremost etiological agent of acute pediatric viral diarrhea worldwide which was a leading cause of death among children less than 5 years of age [1]. A recent study indicated that, in total, 127,539 and 53,559 deaths from diarrhea and RV infection respectively, were reported among children younger than 5 years old in China [2]. RV epidemics are associated with the seasons [3, 4], the sanitary conditions of regions, the conditions people live in, and individual hygiene. RV transmission is mostly by fecal-oral spread in the case of direct and indirect contact [5, 6]. It also can infect many animals, such as calves [7], piglets [8], foals [9], dogs [10], mice, guinea-pigs, sheep, goats, antelopes, monkeys, deer, rabbits and cats [11]. Regardless of two epidemics of severe diarrhea near Lanzhou and Jinzhou in late 1982 and early 1983 [12], RV is a hazard to human [13] and other animals all the time in China.

Usually, RV-induced acute diarrhea is a significant cause of morbidity and mortality in piglets [14]. Previous studies showed that RV-infected pigs have reduced feed intake and weight loss compared to non-infected piglets [15, 16]. The pathology observed in virulent RV-infected in gnotobiotic pigs occurred primarily in intestinal villous epithelial cells with villous atrophy developing as a sequela to infection pathology [17]. Virus particles also can be observed in the small intestine epithelial cells in RV-infected piglets [18].

The pathological lesions caused by RV infection are mainly focused on the changes of intestinal morphology, including the decrease of villi height, the increase of crypt depth, crypt cell production, vacuolization of columnar epithelial cells and border cell fusion [18–20]. So far, several candidate RV vaccines have been assessed for infants and young children, but with variable efficacy [21, 22]. The first licensed RV vaccine, RotaShield, was withdrawn, because it was found to increase the risk of intussusception [23]. However, there was no effective RV vaccine for pig gastroenteritis.

Resveratrol (3, 4’, 5-trihydroxystilbene) is a stilbene and a naturally occurring phytoalexin produced by several plants in response to injury or protecting against microbial infections [24–26]. Previous studies have showed that resveratrol exhibited a number of biological activities, such as antiviral, antibacterial, anti-inflammatory, anticancer, antioxidative, and cardioprotective effects [27]. In our previous study, we found that resveratrol can inhibit duck enteritis virus (DEV) replication *in vitro* [28] and serve as a control measure for DEV infection *in vivo* [29]. In previous studies, resveratrol also was found to possess antiviral activities against HIV [30], SARS [31], HSV [32], varicella-zoster virus [33], and African swine fever virus [34].

In this study, resveratrol dry suspension was used as a feed additive. After pretreatment with resveratrol for 3 weeks, the piglets were challenged with RV. Then, the effects of resveratrol on protecting piglets from damage induced by RV were assessed through clinical diarrhea degree and variations of immunological functions for the purpose of developing a new candidate method for treatment of RV infection.

## 2. Materials and methods

### Ethics statement

All methods and experimental protocols were conducted under the approved guidelines of Sichuan Agriculture University (Chengdu, China) and approved by the ethical committee of the Laboratory Animals Care (Chengdu, China).

### 2.1. Cells, virus and drugs

MA-104 cells were purchased from the China Center for Type Culture Collection (CCTCC, GDC041) and grown in Dulbecco's Modified Eagle Medium (DMEM, HyClone) supplemented with 10% fetal bovine serum (FBS), 100U/ml penicillin, 0.1mg/ml streptomycin. Porcine RV (OSU strain) was purchased from China Veterinary Culture Collection Center (CVCC, AV59). The RV was propagated in MA-104 cells in the presence of 2μg/ml trypsin, and harvested after three freeze-thaw cycles [35]. The titer of the virus was expressed as 50% tissue culture infectious dose per milliliter (TCID_50_/ml) [36]. The resveratrol dry suspension (RDS) was prepared in the Natural Medicine Research Center, Sichuan Agricultural University (Chengdu, China).

### 2.2 Piglets and care

Twenty-eight-day-old piglets were purchased from The Cuirassiers Cialis Animal Husbandry Technology Co., Ltd. (Mianyang, China), and fed in the Basic Veterinary Laboratory of College of Veterinary Medicine, Sichuan Agricultural University (Ya′an, China). The piglets were divided into five groups (six piglets for each) and housed in isolation units. At 32 days, the piglets in three resveratrol-treated groups were administrated with RDS through adding into basal diet at doses of 3 (RDSL), 10 (RDSM) and 30 (RDSH) mg/kg/d, respectively. At 53 days, the piglets in four groups, including untreated with resveratrol but treated with RV group (RVC, Rotavirus Control) and RDS-treated groups, were orally challenged with 4 ml RV supernatant at a dose of 1.0 × 10^6^ TCID_50_/ml. The mock infected group (MI) was orally administered 4 ml DMEM.

At 4 days post-infection (dpi), all animals sacrificed following fasting for a period of 12 hours. The liver was harvested and samples (~5 g) from all animals in each group was rapidly frozen in liquid nitrogen and stored at -80°C until further analysis [37].

### 2.3 Diarrhea score

The criterion of diarrhea score accepted in this study was described as below. Briefly, each stool was awarded a score according to its evaluation of consistency. There were four levels score as follows: 0, normal (no diarrhea); 1, pasty (mild diarrhea); 2, semiliquid (moderate diarrhea); and 3, liquid (severe diarrhea) [38]. There was no diarrhea present in each group prior to infection of the piglets. The diarrhea score of each group was recorded and calculated after RV infection. The scores of 2 and 3 were considered as the onset of diarrhea. The degree of diarrhea among groups was represented as diarrhea index. The parameter was calculated in according with the following formula:

$$\mathrm{Diarrhea}\;\mathrm{index}=\frac{\mathrm{summary}\;\mathrm{of}\;\mathrm{diarrhea}\;\mathrm{scores}}{\mathrm{animal}\;\mathrm{number}\;\mathrm{in}\;\mathrm{the}\;\mathrm{group}\times \mathrm{number}\;\mathrm{of}\;\mathrm{days}\;\mathrm{of}\;\mathrm{trials}}$$

### 2.4 Determination of the peripheral blood T cell subsets

The peripheral lymphocytes were separated from anticoagulant blood. The concentration was diluted to 1.0 × 10^6^ cells/ml with PBS. For two-color staining, peripheral blood mononuclear cells were treated with a mixture of anti-CD3 monoclonal antibody (IgG2a) and anti-CD4 (IgG2b), CD8 (IgG2a) followed by incubation with a mixture of PE-conjugated anti-pig IgG2a (for anti-CD3) and either Alexa Flour 647 mouse anti-pig IgG2b (for anti-CD4), FITC anti-pig IgG2a (for anti-CD8), respectively [39, 40]. Then, flow cytometry analysis was performed on a BD FACSCaliburTM (BD Biosciences, USA). T cell subsets was represented as the percentages of CD3+, CD3+CD4+, CD3+CD8+ in peripheral blood.

### 2.5 Serum and liver antioxidant capacity assay

The liver tissues stored at -80°C were homogenized in PBS (pH = 7.4). Homogenates were centrifuged at 3000 × *g* for 10 min at 4°C [41]. Then the suspensions were used for antioxidant indexes analysis. The protein concentration in livers was estimated with BCA protein assay kit (Beijing Solarbio Science & Technology Co., Ltd., China)

To determine the oxidative injury in piglets induced by RV, the content of malonaldehyde (MDA) and activities of antioxidant enzymes (Superoxide dismutase, SOD; glutathione peroxidase, GSH-Px) in serum and liver were evaluated by using the MDA, SOD and GSH-Px kits, respectively, according to the manufacturer’s instructions (Nanjing Jiancheng Bioengineering Institute, China). The optical densities of each index were recorded using a spectrophotometer (UV-2800A, UNICO, China) at 532nm, 550nm, and 412nm, respectively. The SOD and GSH-Px activities in serum and liver were expressed as U per milligram of protein (U/mg pro).

### 2.6 Serum cytokines assay

Serum samples collected from all piglets at 4 dpi were examined for cytokines, including IFN-γ, TNF-α, IL-4, and IL-10. The concentrations of cytokines in serum were assayed by using enzyme linked immunosorbent assay (ELISA) kits (Shanghai MLBIO Biology Technology Co. Ltd., Shanghai, China) and all processing was as according to manufacturer’s protocols.

### 2.7 Statistical analysis

All data were entered into Excel spreadsheet and imported into SPSS 22.0 for Windows (IBM corporation) for data analysis. The statistical significance in each parameter among the groups was estimated by one-way ANOVA. The independent-samples T test was executed when comparing the significant difference of any parameter between each two groups. All values in the tables are expressed as arithmetic means ± standard deviation (M ± SD), or mean ± standard error of the mean (M ± SEM) in the figures. *P* < 0.05 was considered statistically significant.

## 3. Results

### 3.1 Diarrhea degree

The diarrhea scores of all groups were recorded every day following oral inoculation with RV. The diarrhea index and diarrhea score were showed in Table 1. The diarrhea indexes of the three RDS groups were 1.39, 0.72 and 0.50, respectively. Severe diarrhea developed in RVC group and the diarrhea index was 2.39. No diarrhea in piglets of MI group was found.

**Table 1:** The summary of diarrhea degrees of piglets infected with rotavirus.

Parameters	RVC	RDSL	RDSM	RDSH	MI
Diarrhea index	2.39	1.39	0.72	0.50	0.00
Diarrhea score ≥ 2 (number of piglets)					
2dpi	4/6	2/6	1/6	0/6	0/6
3dpi	6/6	3/6	2/6	1/6	0/6
4dpi	5/6	4/6	1/6	0/6	0/6

RVC, rotavirus control group; RDSL, low dose of resveratrol dry suspension group (3mg/kg/day); RDSM, medium dose of resveratrol dry suspension group (10mg/kg/day); RDSH, high dose of resveratrol dry suspension group (30mg/kg/day); MI, mock infected group.

### 3.2 T lymphocyte subsets

The percentages of CD3+, CD3+CD4+ and CD3+CD8+ among all groups showed no statistical differences (Table 2). RV infection could significantly decrease the CD4+/CD8+ ratio when compared with uninfected group (*P* < 0.05). Treatment with high dose of RDS (RDSH group) could recover the CD4+/CD8+ ratio to normal level (*P* < 0.05).

**Table 2:** The percentages of CD3+, CD3+CD4+, CD3+CD8+in each group (M ± SD, n = 6, unit = %).

Parameters	RVC	RDSL	RDSM	RDSH	MI
CD3+	62.23±3.11	66.23±1.81	62.40±10.76	64.56±8.85	65.46±5.23
CD3+CD4+	23.40±5.06	28.83±3.91	24.23±4.60	30.40±3.73	27.70±4.49
CD3+CD8+	20.53±4.35	21.03±3.93	18.23±4.44	18.60±1.47	18.10±3.47
CD4+/CD8+^1^	1.14±0.13	1.41±0.37	1.39±0.52	1.63±0.19*	1.54±0.14*

RVC, rotavirus control group; RDSL, low dose of resveratrol dry suspension group (3mg/kg/day); RDSM, medium dose of resveratrol dry suspension group (10mg/kg/day); RDSH, high dose of resveratrol dry suspension group (30mg/kg/day); MI, mock infected group.

* Represents significant difference compared with the RVC group (independent-samples t test, *P* < 0.05)

^+^ Represents significant difference compared with the MI group (independent-samples t test, *P* < 0.05)

^1^ The parameter has no unit.

### 3.3 Oxidative stress indexes

**Fig 1:**
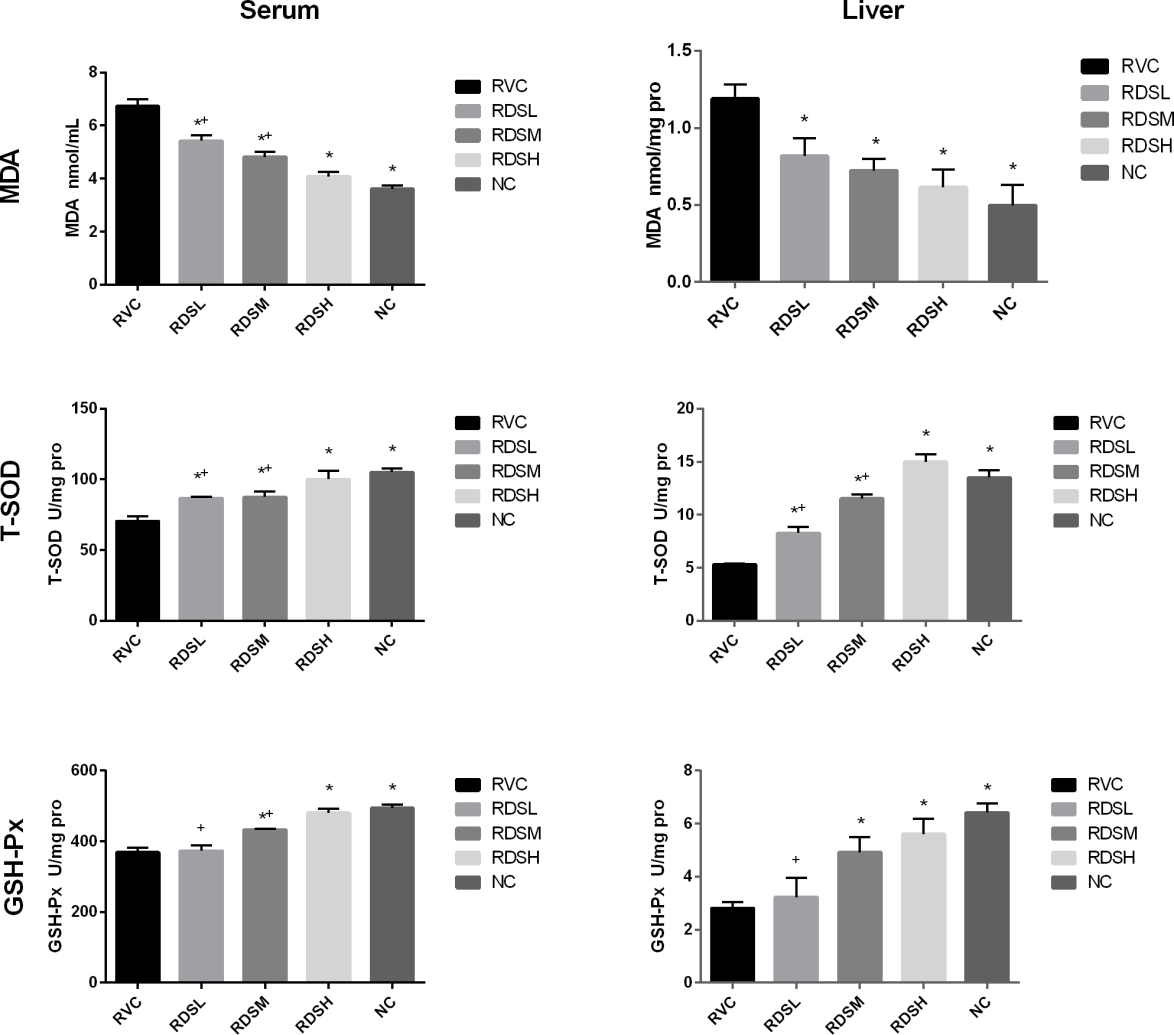
Oxidative stress-associated markers in serum and liver. RVC, rotavirus control group; RDSL, low dose of resveratrol dry suspension group (3mg/kg/day); RDSM, medium dose of resveratrol dry suspension group (10mg/kg/day); RDSH, high dose of resveratrol dry suspension group (30mg/kg/day); MI, mock infected group. * Represents significant difference compared with the RVC group, P < 0.05. ^+^ Represents significant difference compared with the MI group, P < 0.05.

The results of antioxidative levels in serum and livers were showed in Fig 1. In serum, the MDA concentration in the RVC group was increased significantly (*p* < 0.05) compared with that in the MI group. The MDA concentrations in RDS groups were significantly decreased when compared with the RVC group. There was no statistically significant difference between the RDSH group and the MI group on the MDA level. A significant decrease (*p* < 0.05) in SOD and GSH-Px activities were noted in rotavirus control piglets when compared with mock infected piglets. The SOD activities in RDS groups were significantly higher (*p* < 0.05) than that in the RVC group. There was no significant difference in SOD activity between the RDSH group and the MI group. The GSH-Px activities in RDSM and RDSH groups were higher (*p* < 0.05) than that in the RVC group. There was no significant difference between the RDSH group and the MI group.

In liver, the MDA concentration in the RVC group was markedly higher (*p* < 0.05) than that of the MI group. However, the MDA concentrations in RDS groups were significantly decreased (*p* < 0.05) compared to those in the RVC group. There was no significant difference in MDA levels between the RDS groups and the MI group. A significant decrease (*p* < 0.05) in SOD activity was found in the RVC group when compared with the RDS groups and the MI group. There was no significant difference between the RDSH group and the MI group. A significant decrease (*p* < 0.05) in GSH-Px activity was noted in the RVC group compared with RDS groups (RDSM and RDSH) and the MI group. There were no marked differences among RDSM, RDSH and MI groups.

### 3.4 Cytokines in serum

**Fig 2:**
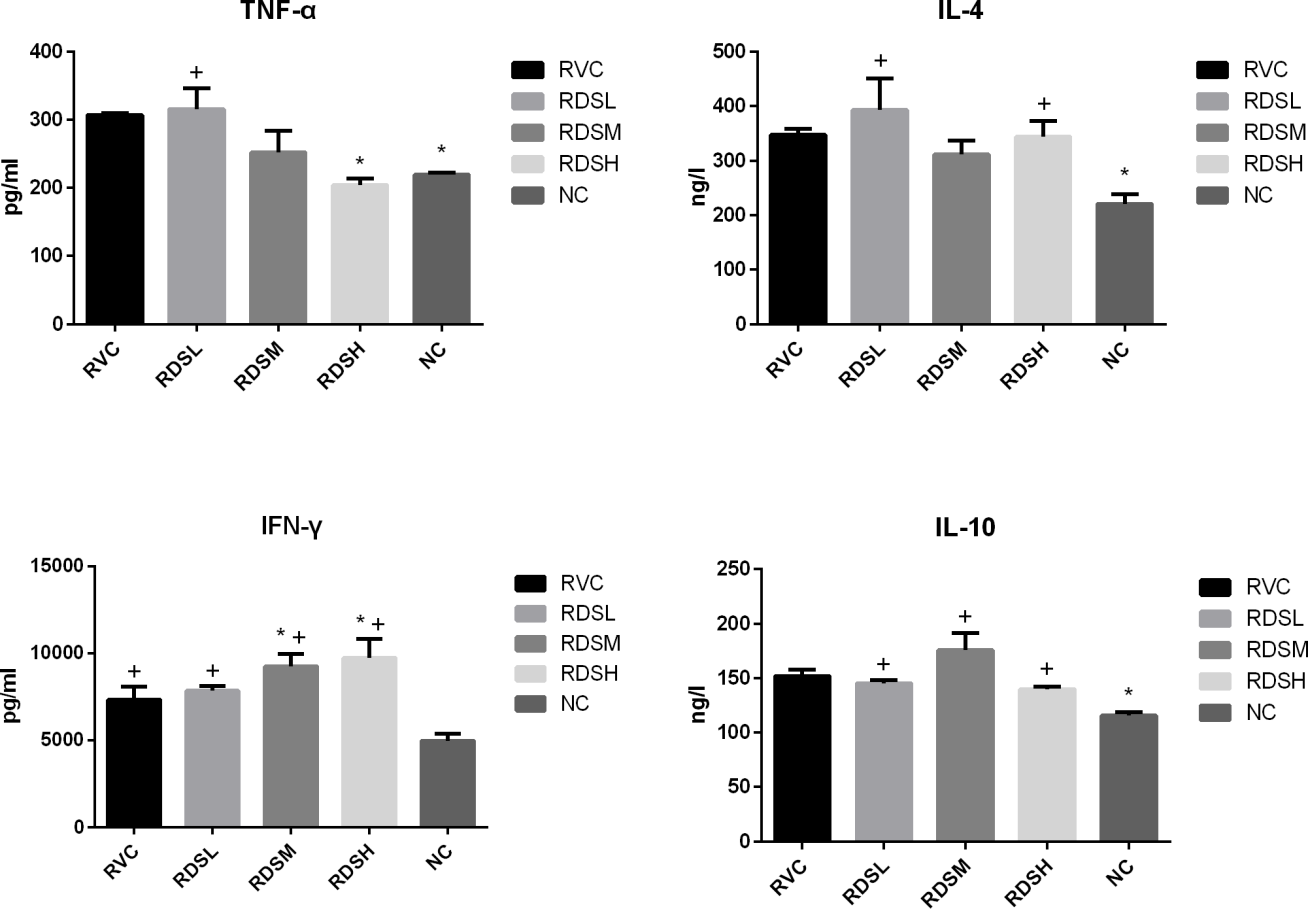
The levels of IFN-γ, TNF-α, IL-4 and IL-10 in serum. RVC, rotavirus control group; RDSL, low dose of resveratrol dry suspension group (3mg/kg/day); RDSM, medium dose of resveratrol dry suspension group (10mg/kg/day); RDSH, high dose of resveratrol dry suspension group (30mg/kg/day); MI, mock infected group. * Represents significant difference compared with the RVC group, P < 0.05. ^+^ Represents significant difference compared with the MI group, P <0.05.

It was reported that resveratrol possessed the ability to inhibit virus replication by regulating cytokine production. Therefore, the concentrations of IFN-γ, TNF-α, IL-4, and IL-10 in serum were detected. The results were showed in Fig 2. The IFN-γ level in the RVC group was significantly higher (*p* < 0.05) in comparison with that in the MI group. When compared with the RVC group, the IFN-γ concentrations in the RDSM and the RDSH groups were statistically increased (*p* < 0.05). The concentration of TNF-α in the RVC group was significantly increased (*p* < 0.05) compared with that in the MI group. With the increase of RDS concentration, the TNF-α level gradually decreased. Compared with the RVC group, the TNF-α concentration in the RDSH group was significantly decreased (*p* < 0.05). In TNF-α production, there were no significant differences between the RDSH group and the MI group. The IL-4 concentration in the RVC group was significantly increased (*p* < 0.05) when compared with that in the MI group. The IL-10 level in the RVC group was significantly higher (*p* < 0.05) than that in the MI group. However, there were no significant differences between the RDS groups and the RVC group.

## 4. Discussion

Resveratrol was notable since the so-called “French Paradox” arose. It was synthesized by many plants in order to resist adverse environmental conditions [42, 43]. For antiviral activity against enterovirus, it was reported that resveratrol could improve Coxsackievirus-induced myocarditis in mice [44] and inhibit enterovirus 71 replication through blocking IKKs/NF-κB signaling pathway [45]. In this study, an important rotavirus (OSU strain) harmful for the pig industry was selected to evaluate the anti-RV efficacy of resveratrol. We found that resveratrol dry suspension could play an important role in inhibition of diarrhea and hepatic injury induced by RV in piglets.

A previous study has been shown that resveratrol significantly attenuated dextran sulphate sodium-induced diarrhea in mice [46]. The diarrhea did not completely disappear after use of RDS for 3 weeks. These results suggested that resveratrol could inhibit diarrhea caused by RV infection in piglets.

Usually, the MDA level was elevated and antioxidant enzymes activity was reduced when the body was in response to oxidative stress. A previous study reported that the concentration of MDA was significantly increased in the serum of piglets suffering from RV enteritis, and the SOD activity was decreased [47]. In mice with dextran sodium sulfate (DSS)-induced ulcerative colitis, resveratrol could significantly decrease MDA concentration in colonic tissue and increase SOD and GSH-Px activities [48]. In our study, similar results were found that resveratrol could decrease MDA concentration and elevate SOD, GSH-Px activities in piglets infected with RV.

IFN-γ is a cytokine that is critical for innate and adaptive immunity against viral and bacterial infections. A previous study showed that it has protective effects against RV infection in children at the early stage [49]. This study showed (Fig 2) that RV infection increases the IFN-γ concentration in the RVC group. After treatment with RDS, the IFN-γ concentration was significantly elevated. TNF-α, a proinflammatory cytokine, could cause damage to the body by inducing inflammation [50]. The lower TNF-α level could protect the organism from inflammatory injury. Our study showed that the TNF-α concentration decreased gradually with the increase of RDS concentration (Fig 2). Based on these results, it suggested that resveratrol could alleviate the inflammation caused by RV infection by decreasing TNF-α concentration, and inhibit RV infection by elevating IFN-γ concentration.

Cytokines after viral infection has important implications for induction of protective immunity. The immune response of children who developed persistent diarrhea following RV infection established that the concentrations of plasma IL-10 and IFN-γ were higher in children with diarrhea compared to uninfected ones [51]. Another previous study showed that cytokines, including IFN-γ, IL-6 and IL-10, played a key role in RV infection and protection against RV disease in children [52]. The levels of IL-4 and IFN-γ were also elevated in gnotobiotic pigs after infection with virulent or attenuated human rotavirus [53]. However, there were no significant differences between the RDS groups and the RVC group on IL-4 and IL-10 levels in this study, proving that the anti-RV effect of resveratrol could not attribute to elevating IL-4 or IL-10 production.

The CD4+/CD8+ ratio is the ratio of T helper cells to cytotoxic T cells. The reduced ratio is associated with reduced resistance to infection [54]. This study found that RV infection could cause a decrease of CD4+/CD8+ ratio. Previous studies showed that the mature CD8+ T cells can clear RV [55, 56]. It is reported that resveratrol supplement increased the amount of CD4+ in rat [57] and the CD4+/CD8+ ratio in chicken [58]. Previous study has declared that the CD4+/CD8+ showed a strong correlation with CD8+ count, but not with CD4+ count [59]. Another study [60] described that CD4+ T cells have an important role in the generation of rotavirus-specific intestinal immunoglobulin A (IgA), which is the principal effector of long-term protection against rotavirus infection, and CD8+ T cells have a role in the timely resolution of primary infection. Therefore, we inferred that the higher CD4+/CD8+ ratio could also play an important role in clearance of RV.

In conclusion, resveratrol dry suspension could suppress diarrhea in piglets infected with RV. It could also reduce the production of proinflammatory cytokines, and maintain the CD4+/CD8+ ratio. Further study should be conducted to evaluate the anti-RV mechanism of resveratrol.
